# Spectrophotometric determination of platelet counts in platelet-rich plasma

**DOI:** 10.1186/s40729-018-0140-8

**Published:** 2018-10-02

**Authors:** Yutaka Kitamura, Masashi Suzuki, Tsuneyuki Tsukioka, Kazushige Isobe, Tetsuhiro Tsujino, Taisuke Watanabe, Takao Watanabe, Hajime Okudera, Koh Nakata, Takaaki Tanaka, Tomoyuki Kawase

**Affiliations:** 1 Tokyo Plastic Dental Society, Kita-ku, Tokyo, Japan; 2 Bioscience Medical Research Center, Niigata University Medical and Dental Hospital, Niigata, Japan; 3 Department of Materials Science and Technology, Niigata University, Niigata, Japan; 4 Division of Oral Bioengineering, Institute of Medicine and Dentistry, Niigata University, Niigata, Japan

**Keywords:** Platelet, Count, Spectrophotometry, Leukocytes, Red blood cells, Quality assurance

## Abstract

**Background:**

Platelet-rich plasma (PRP) is widely used in regenerative dentistry and other medical fields. However, its effectiveness has often been questioned. For better evaluation, the quality of individual PRP preparations should be assured prior to use. We proposed a spectrophotometric method for determination of platelet counts and validated its applicability using two types of PRP preparations.

**Methods:**

Blood samples were obtained from healthy male volunteers and pure PRP (P-PRP) and leukocytes-rich PRP (L-PRP) were prepared using the double-spin method. In serial dilutions, platelet counts in P-PRP and L-PRP were determined using an automated hematology analyzer and a compact spectrophotometer. For validation, P-PRP and L-PRP independently prepared by three well-trained operators were used for comparison of the calculated and measured platelet counts.

**Results:**

In the two types of PRP samples evaluated, platelet counts were almost equal and greater amount of both white blood cells (WBCs) and red blood cells (RBCs) were included in L-PRP preparations. The calibration curve obtained from serially diluted P-PRP showed a strong correlation (*R*^2^ = 0.995), whereas that of L-PRP was relatively weaker (*R*^2^ = 0.975). In validation testing, the scatter plot of the calculated platelet counts versus the measured values showed a strong correlation in P-PRP (*R*^2^ = 0.671), whereas that of L-PRP showed a much weaker correlation (*R*^2^ = 0.0605).

**Conclusions:**

This method can precisely determine platelet counts in PRP preparations when the inclusion of WBCs or RBCs is minimized. Therefore, we recommend that clinicians use this method for quality assurance of individual PRP preparations.

## Background

Almost two decades have passed since platelet concentrates, such as platelet-rich plasma (PRP), were first introduced to the field of regenerative medicine by Marx et al. [1]. To date, PRP has been modified to create different variations and has increasingly been used in various fields of regenerative therapy around the world. However, negative data obtained from clinical applications of PRP have often been reported, leading to controversy regarding the predictability of PRP therapy [2]. Especially in cases of skeletal regeneration, the efficacy of PRP has been controversial [3–9].

One possible major reason behind this debate is the lack of large controlled clinical trials [2] or randomized clinical trials. Because there is no consensus regarding the indications and contraindications for PRP therapy, it is theoretically difficult to design appropriate experiments. In addition, there are no generally accepted guidelines on how to evaluate the condition of application sites. The second major reason, which has frequently been used as a possible explanation (actually, an “excuse”) for unexpected clinical results in many clinical case reports, is individual difference. This is highly conceivable, but not convincingly supported by scientific evidence in individual cases. The third major reason is the lack of consensus regarding PRP preparation protocols [2]. Recent advances in the development of various automated preparation devices and kits are expected to reduce not only the labor of the operator but also technique-dependent variation of PRP quality. However, it should be noted that these devices cannot standardize PRP quality. In other words, it is not guaranteed that the quality of individual PRP preparations depends specifically on individual preparation devices. In fact, it is well-known that PRP and its derivatives prepared using the same devices do not necessarily induce similar clinical results.

In Japan, a new regulatory framework for PRP therapy was established in 2014. However, no evaluation indexes for PRP quality, except for aseptic handling to ensure sterility, are indicated in the regulations. In our recent review article [10], we highlighted the necessity of PRP quality indexes. The primary index is platelet counts. Specifically, it is best to check platelet counts prior to use. To assess PRP quality in clotted PRP derivatives, such as platelet-rich fibrin (PRF), we recently developed a direct counting method for platelets contained in fibrin clots [11]. However, only a few clinicians possess automated hematology analyzers (AHAs) or similar electronic devices that can be used to determine platelet counts accurately without bias or technical error.

In this study, we focused on the possibility of spectrophotometric determination and validated the applicability of the proposed method on platelet counts in PRP preparations. This idea was based on bacterial cell counting [12] and a similar challenge was reported in 1992 [13]. However, this optical method has not been further modified for PRP as a grafting material for regenerative therapy in accordance with the policy of quality assurance. Based on the count of white blood cells (WBCs) and red blood cells (RBCs) included in PRP preparations, we categorized PRP preparations into two types as follows: pure PRP (P-PRP) and leukocyte-rich PRP (L-PRP) [14–16]. As not only platelets but also WBCs are concentrated in L-PRP, we hypothesized that the inclusion of WBCs at higher levels could markedly interfere with this spectrophotometric determination. As predicted, we validated the applicability of our proposed method by precisely determining platelet counts in P-PRP, but not L-PRP.

## Methods

### Preparation of P-PRP and L-PRP

Blood samples were collected from 11 non-smoking healthy male volunteers aged 33 to 69 years. The study design and consent forms for all the procedures were approved by the ethics committee for human participants at the Niigata University School of Medicine (Niigata, Japan) in accordance with the Helsinki Declaration of 1964 as revised in 2013.

Peripheral blood (~ 9 mL) was collected into plastic vacuum plain blood collection tubes (Neotube; NIPRO, Osaka, Japan) containing 1 mL of the A-formulation of acid-citrate-dextrose (ACD-A; Terumo, Tokyo, Japan). The whole-blood samples were stored using a rotating agitator at ambient temperature and were used within 36 h. The whole-blood samples were centrifuged at 533×*g* for 10 min (first low-speed spin). For P-PRP preparation, the upper plasma fraction, which was approximately 2 mm beyond the interface between the plasma and RBC fractions, was transferred into 2-mL sample tubes for the second high-speed spin (2656×*g*, 5 min). For L-PRP preparation, the upper plasma fraction was transferred along with a buffy coat and the surface of the RBC fraction for the second spin. Prior to the second spin, 0.5 μg/mL prostaglandin E_1_ (PGE_1_) (Wako Pure Chemicals, Osaka, Japan) was added to each sample to prevent platelet aggregation. After centrifugation, 50–70% of the supernatant (PPP) was removed, and platelets (and other blood cells, if any) were resuspended in the remaining PPP fraction.

The numbers of platelets and other blood cells in the whole-blood samples and PRP preparations were determined using an AHA (pocH 100iV, Sysmex, Kobe, Japan).

### Spectrophotometric determination of platelet counts and calibration curves

**Fig. 1:**
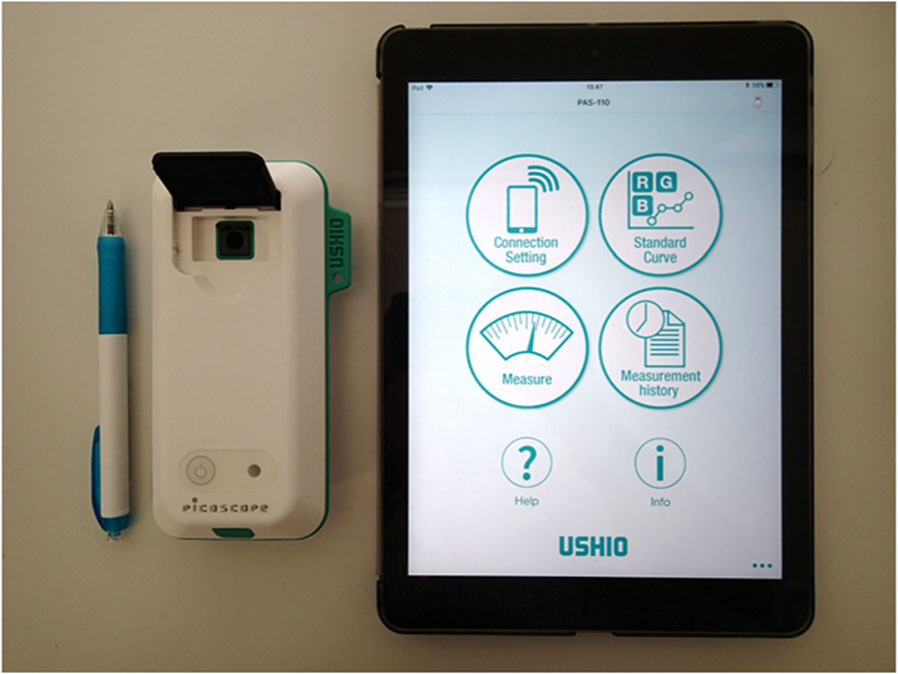
A compact SPM with its remote controller installed on an iPad Air. iPhones and other Android devices can be used instead of the iPad Air

P-PRP and L-PRP preparations were serially diluted with the corresponding amount of PPP. The series of P-PRP and L-PRP dilutions were first subjected to measurement using the AHA and subsequently subjected to measurement with a compact scanning probe microscope (SPM; PiCOSCOPE, Ushio Inc., Tokyo, Japan) (Fig. 1). The SPM can be operated by remote control through a specific application installed on smart devices, including the iPad Air (Apple, Cupertino, CA, USA). PRP samples were transferred into 0.2 mL highly transparent PCR tubes (Nippon Genetics Co., Ltd., Tokyo, Japan) and were measured at 615 nm (range of wavelength 570–660 nm).

Using the data obtained with both the AHA and SPM, scattered plots were created to examine correlations and obtain formulas to calculate platelet counts.

### Validation testing

P-PRP and L-PRP preparations were independently prepared from the 11 donors by three well-trained operators. Platelet counts were first determined using the AHA and aliquots of the PRP preparations were measured using the SPM. Platelet counts were calculated with the appropriate formulas and were compared with the measured platelet counts.

### Statistical analysis

The data are expressed as mean ± standard deviation (SD). For two-group comparisons, statistical analyses were conducted to compare the mean values using the Student’s *t* test (SigmaPlot 12.5; Systat Software, Inc., San Jose, CA, USA). *P* values of < 0.05 were considered statistically significant. The strength of a linear association between measured platelet counts and absorbance values was evaluated using the Pearson correlation coefficient (*R*). Based on these data, we obtained formulas for calculating platelet counts using absorbance values. Additionally, possible correlations between platelets and RBCs or WBCs and those between measured and calculated platelet counts were also evaluated using the Pearson correlation coefficient.

## Results

**Fig. 2:**
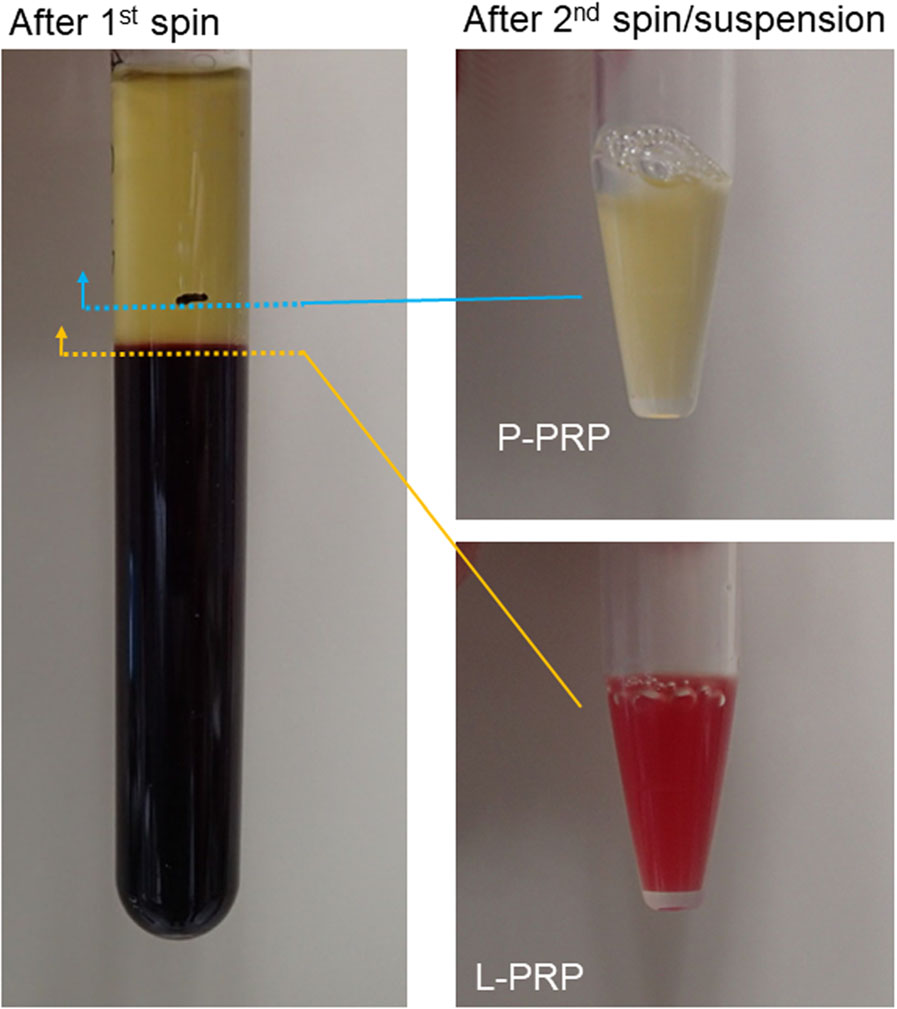
The appearance of blood sampled after gravity fractionation and the resulting P-PRP and L-PRP. In the first low-speed spin, samples were centrifuged for 10 min at 533×*g*. For P-PRP preparation, the upper plasma fraction, which was 2 mm beyond the interface between plasma and RBC fractions, was transferred into sample tubes for the second high-speed spin (2656×*g*, 5 min). In contrast, for L-PRP preparation, the upper plasma fraction including the buffy coat and the surface of the RBC fraction was used for the second spin. The supernatant (PPP) was excluded by 50–70%, and platelets were resuspended in the remaining PPP fraction

The appearance of the blood-collection tube after the first low-speed spin and representative P-PRP and L-PRP preparations after the second high-speed spin and subsequent re-suspension are shown in Fig. 2. Although low-speed spinning did not result in the formation of a clear buffy coat in the interface between the plasma and RBC fractions, the buffy coat corresponding to the plasma was not included in the second spin for P-PRP preparation. Therefore, the resulting P-PRP was light yellow in color, not reddish. In contrast, for the L-PRP preparation, the buffy coat and the surface of the RBC fraction just below the interface were included in the second spin. The inclusion of significant amounts of RBC turned the L-PRP red. The strength of this color was variable depending on the operators’ pipetting skills; however, L-PRP preparations were more or less reddish when the maximum amount of platelets was recovered.

**Fig. 3:**
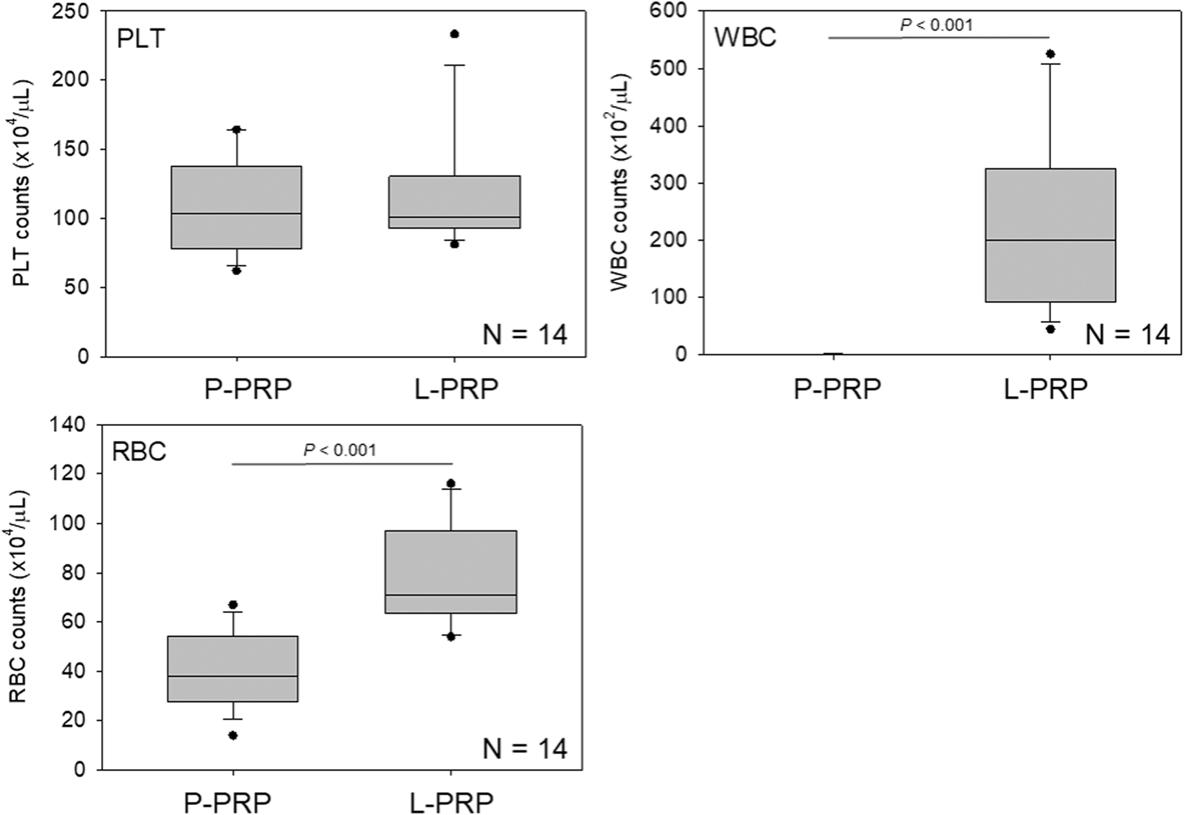
Counts of platelets (PLT), WBCs, and RBCs in P-PRP and L-PRP preparations prepared for calibration curves. *N* = 14 for each type of PRP

To characterize both the P-PRP and L-PRP preparations used for the calibration curves, blood cells were counted using an AHA (Fig. 3). For platelet counts, there was no significant difference between the two types of PRP. For WBC and RBC counts, in contrast, L-PRP contained significantly more WBCs and RBCs than P-PRP.

**Fig. 4:**
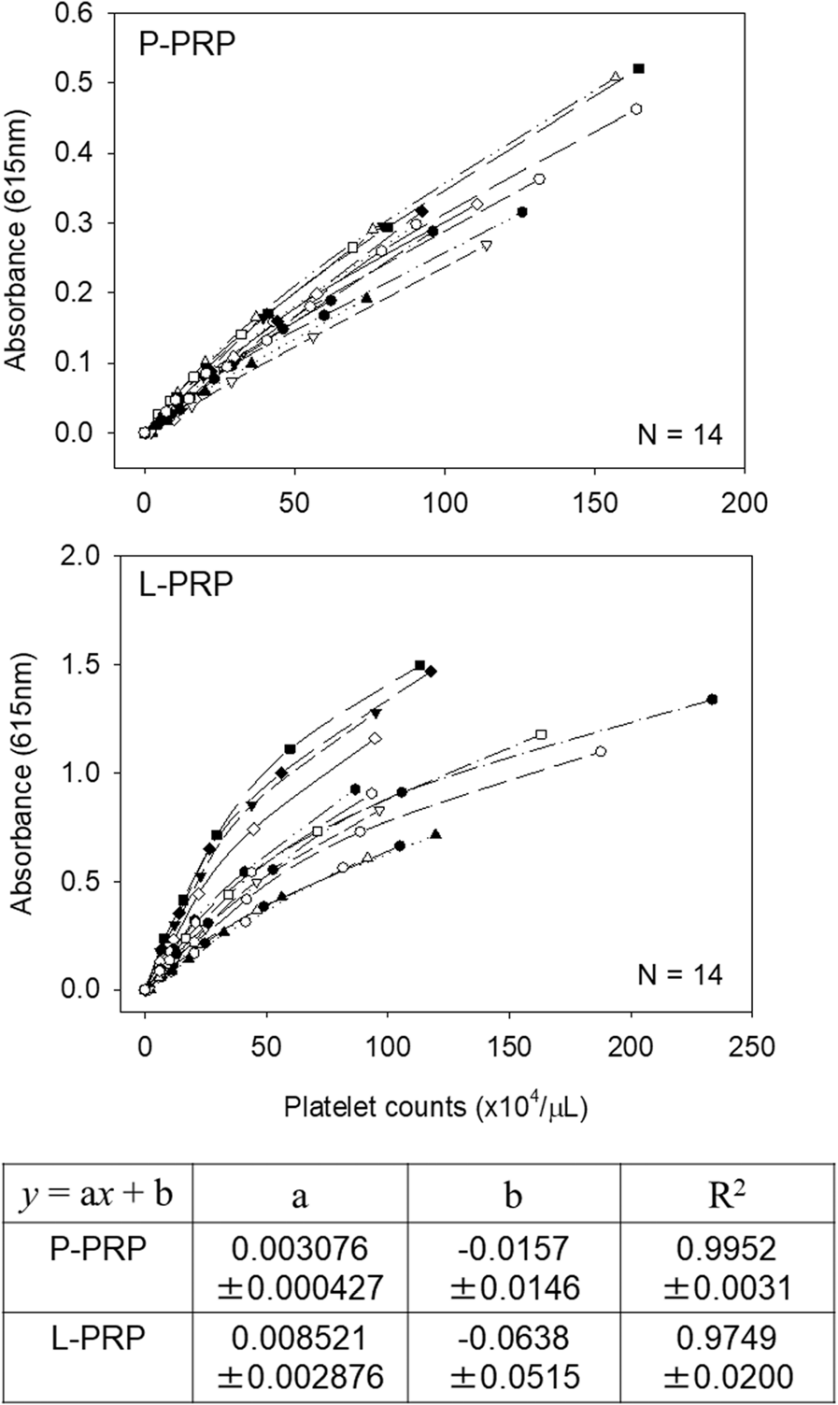
Calibration curves of measured platelet counts versus absorbance in P-PRP and L-PRP preparations. The samples were serially diluted by PPP, and the platelet counts were determined using an AHA and SPM. *N* = 14 for each type of PRP

The samples were serially diluted, and platelets in individual dilutions were counted using the AHA. In parallel, the absorbance of each sample was measured with the SPM. The resulting calibration curves for P-PRP and L-PRP are shown in Fig. 4. Compared with P-PRP, the calibration curves for L-PRP varied with the samples and appeared generally inappropriate for linear regression. The calibration curve for P-PRP was expressed as “*y* = 0.00308*x* − 0.0157,” while that of L-PRP was “*y* = 0.00852*x* − 0.638.” The SD values for both the slope and intercept values were much higher in L-PRP. In addition, the *R*^2^ value (coefficient of determination) for the linear regression of P-PRP was 0.995, while that of L-PRP was a little lower than that of P-PRP, 0.975, with almost 6.5-times higher SD values.

**Fig. 5:**
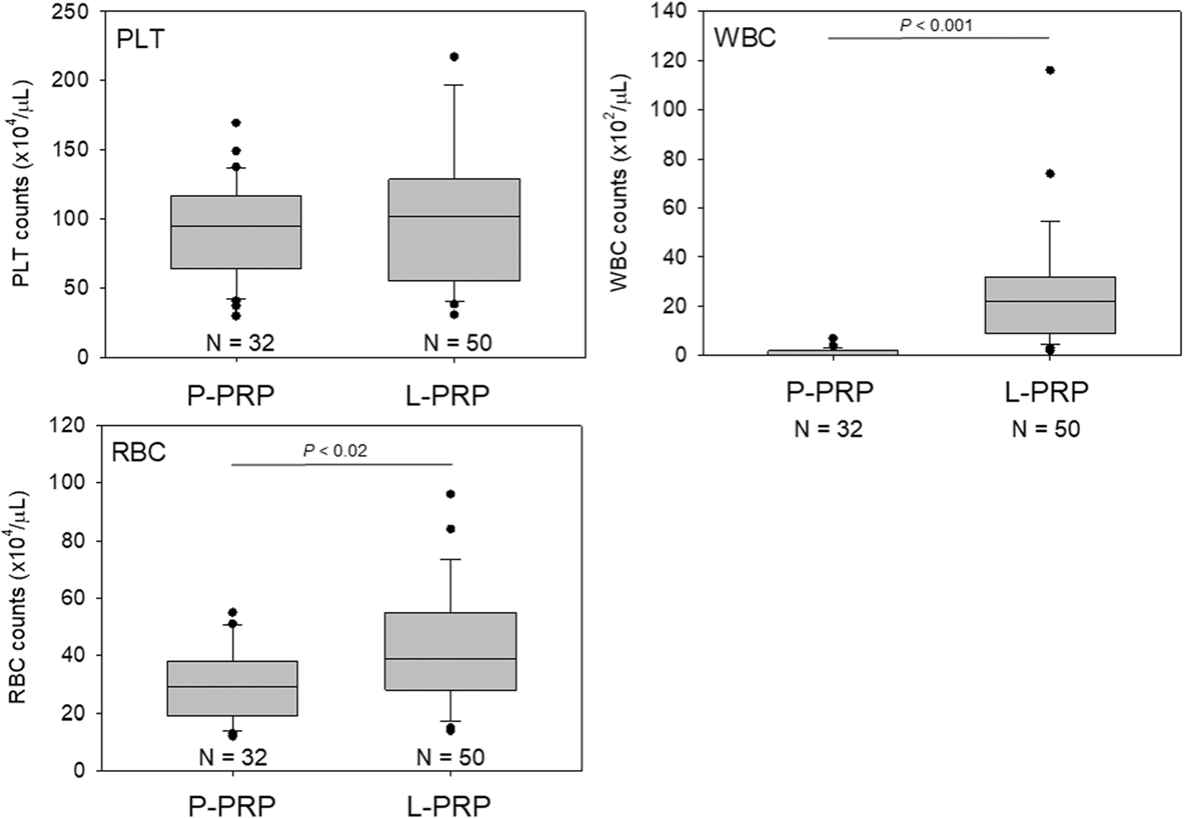
Counts of platelets (PLT), WBCs, and RBCs in P-PRP and L-PRP preparations prepared for validation testing. *N* = 32 and 50 for P-PRP and L-PRP, respectively

**Fig. 6:**
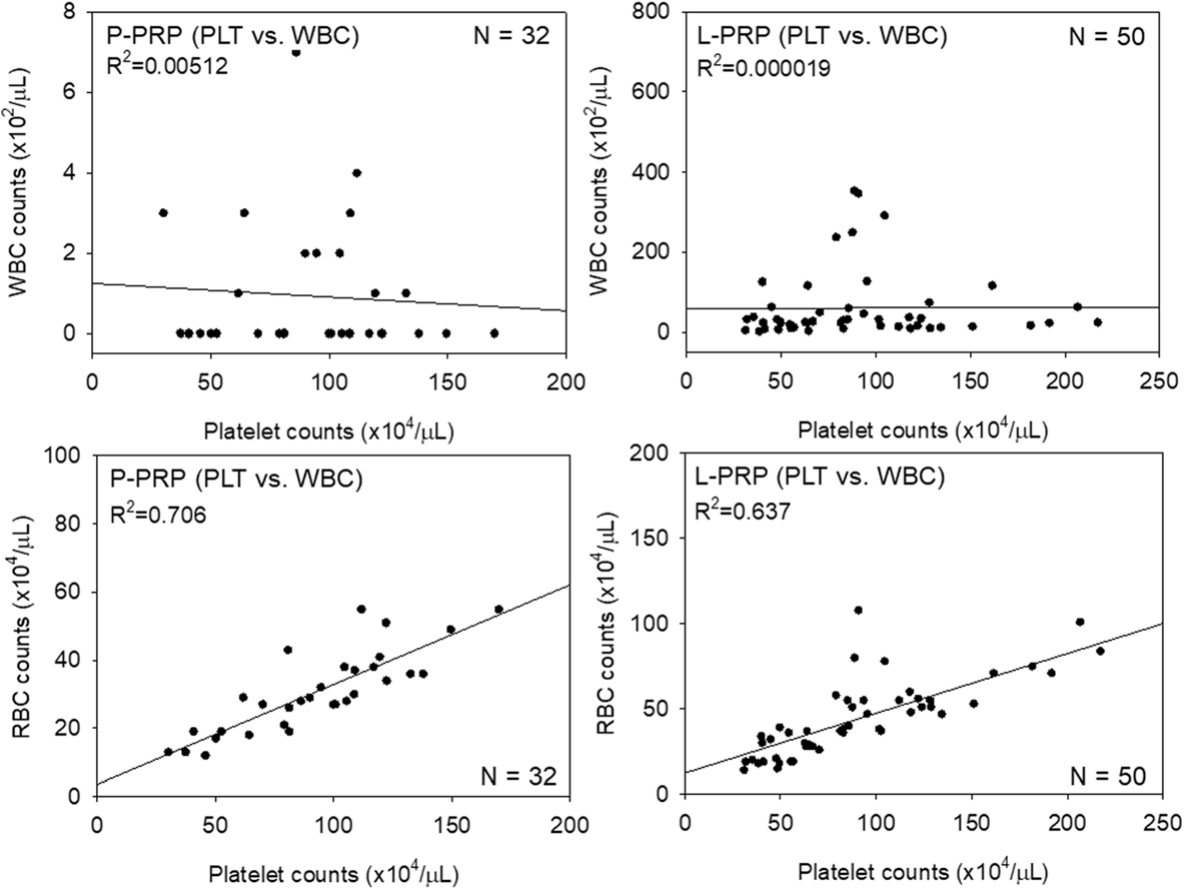
Scatter plots representing possible correlations between platelet (PLT) and WBC counts and between platelet and RBC counts in P-PRP and L-PRP preparations. Note: strong positive correlations were observed between platelets and RBC in both PRP types. *N* = 32 and 50 for P-PRP and L-PRP, respectively

For validation of these calibration curves, P-PRP and L-PRP preparations prepared by three independent operators were employed. Blood cell counts are shown in Fig. 5. As observed in the calibration curves for the samples, significant differences were found in WBC and RBC counts, but not in platelet counts, between the P-PRP and L-PRP preparations. Correlations between platelet counts and WBC or RBC counts are shown in Fig. 6. Unexpectedly, strong positive correlations were observed only between platelet and RBC counts, but not between platelet and WBC counts, in both types of PRP preparations.

**Fig. 7:**
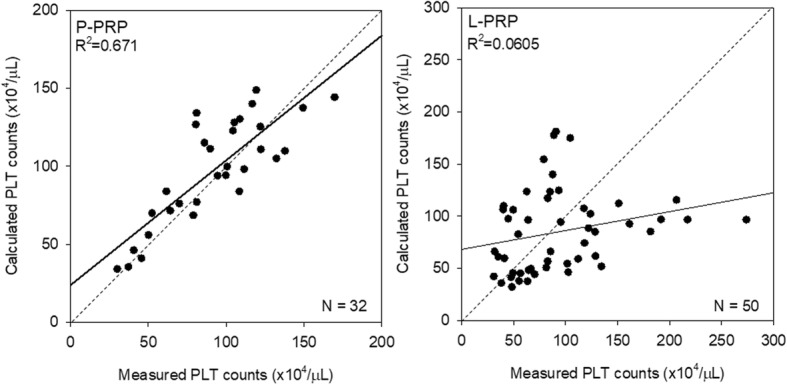
Scatter plots representing correlations between measured and calculated platelet counts in P-PRP and L-PRP preparations. Note: a strong correlation was observed only in P-PRP. *N* = 32 and 50 for P-PRP and L-PRP, respectively

Measured versus calculated platelet counts are plotted in Fig. 7. In P-PRP preparations, the ratio of calculated platelet counts to the measured values was 108.6 ± 22.0%, whereas in L-PRP preparations, the ratio was 110.4 ± 64.0%. The discrepancy of SD values was reflected more clearly in the difference of *R*^2^ values (0.671 vs. 0.0605).

## Discussion

Since determination of bacterial cell number is a fundamental procedure in the field of microbiology, several methods have been developed and widely employed depending on the purpose of cell counting. SPM is one of the common methods used to estimate bacterial load [12]. The advantage of SPM is speed and convenience without additional preparation steps. On the other hand, the limitations are the inability to distinguish live bacteria from dead bacteria and a relatively narrow range of detection (10^8^–10^10^ bacteria/mL) [12].

A wide range of detection is not required for platelet counting in PRP preparations unlike in bacterial cell counting. However, it is more difficult to recognize platelets in PRP preparations compared to bacteria because WBCs and RBCs can more or less be included, especially when the buffy coat is included in the second spin. Lee and Tarassenko were probably inspired by the bacterial cell count and first reported the optical determination method for platelet counts [13]. However, the shortcomings of this method are that the range of RBC counts (0–3 × 10^4^/μL) is set below the RBC range (30–40 × 10^4^/μL in average) of P-PRP and that WBCs were not taken into consideration.

To solve this problem, in this study, we separated PRP preparations into two types (i.e., P-PRP and L-PRP) for evaluation and successfully validated the spectrophotometric method in P-PRP preparations. In contrast, the accuracy of this method was lower than expected in L-PRP preparations, which is reflected in the difference in the coefficient values (Fig. 4). The striking difference between P-PRP and L-PRP could be attributed to the inclusion of WBCs rather than RBCs in L-PRP as RBCs were also included in P-PRP with higher platelet counts. We speculate that WBCs were the primary factor responsible for lowering the performance and that they can disrupt light transparency more effectively than can RBCs; this is because WBCs are spherical, nucleated, and larger than disk-shaped RBCs and because the absorbance of hemoglobin contained in RBCs decreases beyond 600 nm [17] (cf., 615 nm, the peak wavelength used here). Besides counts, the size distribution of WBCs depends on individual donors. Hence, the ratios of large WBCs (e.g., neutrophils) to small WBCs (e.g., lymphocytes) widely vary across individuals, especially when they suffer from certain types of diseases, such as cancers, cardiovascular diseases, and pulmonary diseases [18–21].

Another limitation is the color of plasma. In terms of color, blood samples obtained from the donors participating in this study were light yellow and could be evaluated as “normal.” However, we have sometimes encountered colored plasma samples in clinical practice. For example, when blood triglyceride levels are high, the plasma turns milky white or turbid [22–24]. Hemolytic plasma looks reddish, while icteric plasma appears yellow. When the degree of color change is not severe and when the transparency is maintained, the data may be compensated by the absorbance of PPP. However, in this case, we recommend the use of an AHA for accurate determination of the platelet counts.

We should discuss briefly how clinicians can perform quality assurance for individual PRP preparations. As described elsewhere [10, 25], PRP quality is evaluated mainly based on two major points: sterility and efficacy. Recent advances in PCR technology enable clinicians to quickly assess the contamination of targeted bacteria and mycoplasmas [26] in clinical settings. However, clinicians may require a well-trained operator for this kind of sterility testing. The current regulatory framework for PRP therapy in Japan requires clinicians to prepare PRP on a clean bench [10]. Therefore, as long as blood samples are handled aseptically, the resulting PRP preparations are evaluated as sterile.

As for efficacy, regardless of the assay system, several hours or days are required to complete efficacy testing. Even if it takes only several hours, unfortunately, this delay is not beneficial to many patients and is not suitable for on-site preparation and immediate use in autologous PRP therapy. The only exception is platelet counting, which takes only a few minutes with the use of an AHA. However, it is a problem that the conventional form of this device is $10,000 or higher and requires installation space (500 × 500 mm at least). In contrast, the compact SPM used in this study costs only $800 and can be stored in a drawer. Therefore, despite several limitations, this compact SPM would be useful for fundamental quality assurance as well as for the examination of possible correlations between platelet counts and clinical outcomes.

Consistent with the clinical significance of platelet counting, several studies have reported that the platelet concentration is the most reliable criterion for the regenerative ability of PRP [27, 28] because platelets increase the number of anabolic signaling molecules. Conversely, as WBCs increase the number of catabolic signaling molecules, the quality of PRP can, perhaps, be considerably altered depending on the levels of WBCs included in PRP [29]. Despite functioning to clean wounds and prevent infection, WBCs, particularly phagocytic leukocytes, have been reported to produce matrix metalloproteinases (MMPs), oxygen and nitrogen reactive species (free radicals), and proinflammatory cytokines, which could adversely affect the stem cell behavior and, consequently, tissue regeneration [27, 30, 31]. This finding is evidenced by the fact that L-PRP induces inferior effects on the bone and cartilage regeneration compared with P-PRP [32, 33], indicating that P-PRP is, perhaps, more suitable than L-PRP in the field of regenerative dentistry. Hence, although working only in P-PRP, our spectrophotometric method would be of great use in assuring the quality of individual PRP preparations in the dental setting.

## Conclusions

In normal blood samples composed of light yellow plasma, spectrophotometric determination of platelet counts would be useful for quality assurance of individual PRP preparations. For accurate determination, however, operators should handle samples with care to minimize the inclusion of WBCs and RBCs in PRP preparations.

## Abbreviations

ACD: Acid-citrate-dextrose solution
AHA: Automated hematology analyzer
L-PRP: Leukocyte-rich PRP
PGE_1_: Prostaglandin E_1_
PPP: Platelet-poor plasma
PRF: Platelet-rich fibrin
PRP: Platelet-rich plasma
P-PRP: Pure-PRP
RBC: Red blood cell
SD: Standard deviation
SPM: Spectrophotometer
WBC: Leukocyte
